# Study on the Effective Material Basis and Mechanism of Traditional Chinese Medicine Prescription (QJC) Against Stress Diarrhea in Mice

**DOI:** 10.3389/fvets.2021.724491

**Published:** 2021-10-04

**Authors:** Yuefeng Zhang, Fei Yu, Jingyou Hao, Eliphaz Nsabimana, Yanru Wei, Xiaohan Chang, Chang Liu, Xiaozhen Wang, Yanhua Li

**Affiliations:** ^1^Heilongjiang Key Laboratory for Animal Disease Control and Pharmaceutical Development, College of Veterinary Medicine, Northeast Agricultural University, Harbin, China; ^2^Harbin Lvda Sheng Animal Medicine Manufacture Co., Ltd., Harbin, China; ^3^Harbin Herb and Herd Bio-Technology Co., Ltd., Harbin, China

**Keywords:** traditional Chinese medicine, active components, network pharmacology, stress-induced diarrhea, PI3K–Akt signaling pathway

## Abstract

Stress diarrhea is a major challenge for weaned piglets and restricts pig production efficiency and incurs massive economic losses. A traditional Chinese medicine prescription (QJC) composed of *Astragalus propinquus* Schischkin (HQ), *Zingiber officinale* Roscoe (SJ), and *Plantago asiatica* L. (CQC) has been developed by our laboratory and shows marked anti-stress diarrhea effect. However, the active compounds, potential targets, and mechanism of this effect remain unclear and warrant further investigation. In our study, we verified the bioactive compounds of QJC and relevant mechanisms underlying the anti-stress diarrhea effect through network pharmacology and *in vivo* experimental studies. After establishing a successful stress-induced diarrhea model, histomorphology of intestinal mucosa was studied, and Quantitative real-time PCR (RT-qPCR) probe was used for the phosphoinositide 3-kinase (PI3K)–Akt signaling pathway to verify the therapeutic effect of QJC on diarrhea. First, using the network pharmacology approach, we identified 35 active components and 130 Kyoto Encyclopedia of Genes and Genomes (KEGG) pathways in QJC. From among these, we speculated that quercetin, luteolin, kaempferol, scutellarein, and stigmasterol were the main bioactive compounds and assumed that the anti-diarrhea effect of QJC was related to the PI3K–Akt signaling pathway. The RT-qPCR indicated that QJC and its bioactive components increased the expression levels of *PI3K* and *Akt*, inhibited the expression of *phosphatase and tensin homolog* (*PTEN*), and activated the PI3K–Akt signaling pathway to relieve stress-induced diarrhea. Furthermore, we found that QJC alleviated the pathological condition of small intestine tissue and improved the integrity of the intestinal barrier. Taken together, our study showed that the traditional Chinese medicine QJC, quercetin, luteolin, kaempferol, scutellarein, and stigmasterol alleviated the pathological condition of small intestine tissue and relieved stress-induced diarrhea by increasing the expression levels of *PI3K* and *Akt* and inhibiting the expression levels of *PTEN*.

## Introduction

With continuous developments in the pig industry, early weaning of piglets has become a general consensus (1); the period is usually accompanied by diarrhea, which brings huge economic losses to the pig industry (2). During weaning, piglets are exposed to and must quickly adapt to various psychological, environmental, and physiological stressors (1), all of which are associated with weaning-related stress, which leads to changes in intestinal structure and function as well as imbalances in intestinal microbiota (3), eventually leading to post-weaning stress diarrhea. At present, dietary intervention is the most common method to alleviate weaning stress diarrhea (4, 5), and plant extracts can improve the growth performance, antioxidant capacity, and immunity of livestock and poultry to prevent various diseases (6, 7).

**Figure 1:**
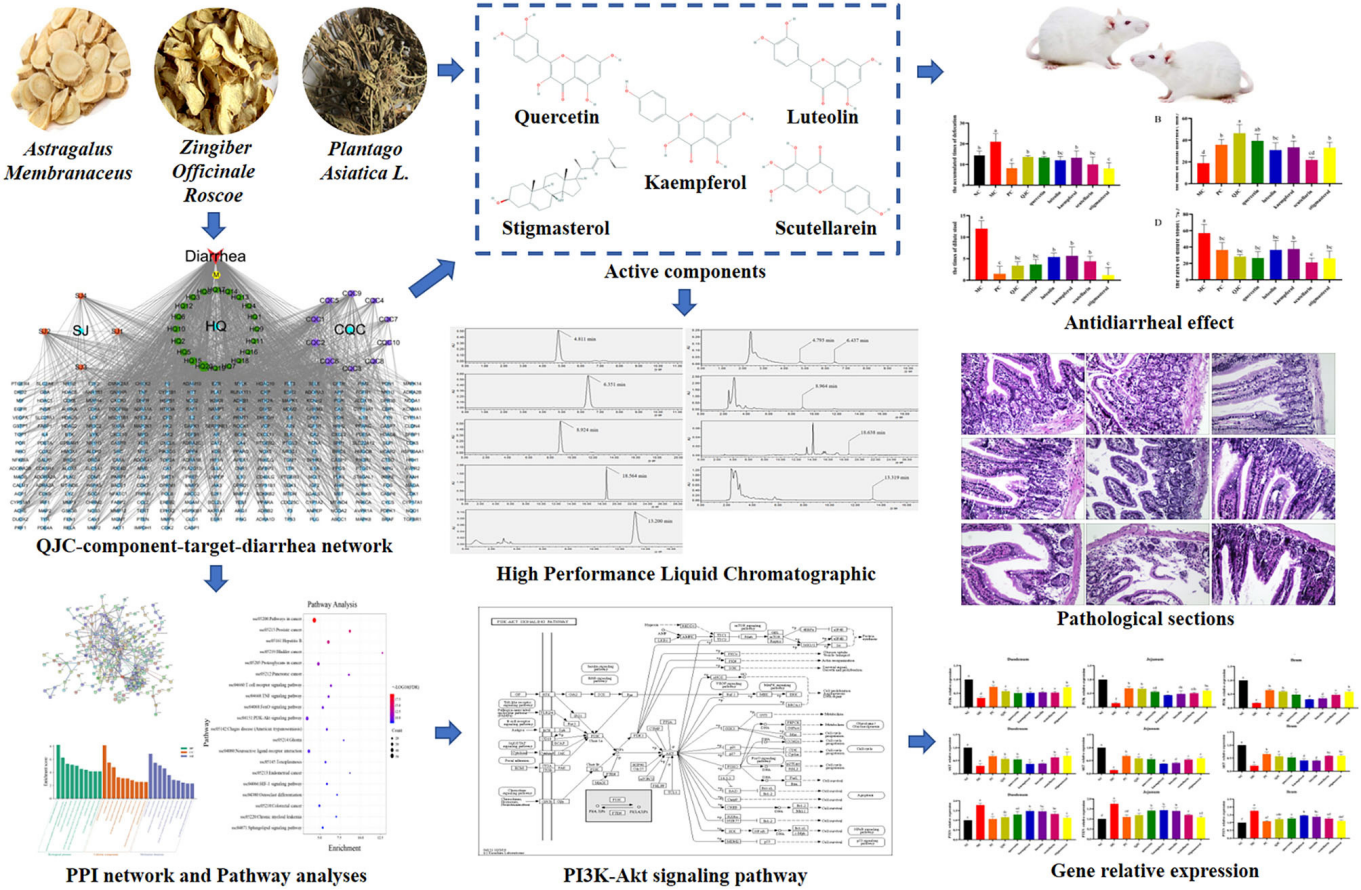
The experimental flowchart.

Previous studies in our laboratory have shown that *Plantago asiatica* L. (CQC), an important medicinal plant, can effectively treat diarrhea (8). On the basis of the previous research results, two kinds of traditional Chinese medicine (TCM), *Astragalus propinquus* Schischkin (HQ), and *Zingiber officinale* Roscoe (SJ), were added to this study. HQ reportedly activates T cells and natural killer cells by stimulating the production of macrophages and immunoglobulin, which have immunity-enhancing effects (9), thus reducing the incidence of diarrhea in weaned piglets, preventing harmful flora from growing in the intestine, enhancing piglet immunity, and improving weaning piglet production performance (10). Besides, SJ reportedly restores intestinal microbiota and intestinal barrier function (11), and its extract 6-gingerol can block the secretion of tumor necrosis factor (TNF)-α, interleukin (IL)-6, and inducible nitric oxide synthase (iNOS) in the cecal cells of diseased mice (12). Moreover, 6-gingerol can protect mice from ulcerative colitis (UC) by inhibiting oxidative stress and pro-inflammatory mediators (13). In addition, the addition of CQC in the feed of weaned piglets positively impacts their intestinal microbiota (14). Early reports suggested that CQC exerts its anti-diarrhea effect by effecting Na^+^/K^+^-ATPase and creatine kinase activities and the Na^+^/K^+^ concentration (8). However, the relationship between the active compound and the mechanism of QJC in view of the anti-diarrhea effect remains unclear, and further investigation is needed. Presently, many methods are used to study the pharmacodynamic material basis, such as using high-throughput screening, metabolomics, and network pharmacology (15–17). It is well known that network pharmacology provides a holistic approach to search and comprehend the actions of traditional medicine (18–20). Recent studies have shown that TCM has the characteristics of multi-component, multi-target, and multi-pathway (21); network pharmacology has similar features. Therefore, we used the network pharmacology approach to predict the bioactive ingredients of QJC and revealed the mechanism of alleviating diarrhea through an *in vivo* experimental study. The experimental flowchart of this study is shown in Figure 1.

## Materials and Methods

### Network Pharmacology-Based Analysis

#### Active Compounds and Corresponding Target Collection

All of the chemical ingredients of QJC were collected from Traditional Chinese Medicine Systems Pharmacology Database and Analysis Platform (TCMSP; https://tcmspw.com/tcmsp.php). Preliminary screening was based on the characteristics of absorption, distribution, metabolism, and excretion (ADME), oral availability (OB) ≥30%, and drug-likeness (DL) ≥0.18. Among these, OB reveals the ADME process and represents the proportion of oral drug absorbed into the systemic circulation (22). Drug-likeness is applied to evaluate the selection criteria for these “drug-like” compounds (23). To obtain as many targets as possible, we transformed the structures of the candidate compounds into 2D SDF structural forms by PubChem (https://pubchem.ncbi.nlm.nih.gov/) and used Swiss Target Prediction (http://www.swisstargetprediction.ch/) to calculate targets. Then, taking all these target genes as queries, genes from *Sus scrofa* (pig) were preserved by searching the Uniprot database (https://www.uniprot.org/) (15).

#### Candidate Target Collection

“Diarrhea” was used as the keyword, and targets were downloaded from OMIM (http://www.omim.org), DisGeNET (https://www.disgenet.org/), and Gene Cards (https://www.genecards.org/). Then, they were merged, and duplications were removed. All genes were corrected on Uniprot with *S. scrofa* (pig) as the target animal.

#### Target Prediction

Interactive Venn online tools (http://www.interactivenn.net/) were used to acquire the common targets of “QJC-component-target-diarrhea” for predicting the potential QJC targets for alleviating piglet diarrhea. Then, we visualized the “QJC-component-target-diarrhea” network using Cytoscape 3.7.2 (http://www.cytoscape.org/) and analyzed the topology of the network. In our study, degree was selected as a measure of node importance: if the degree of the nodes is higher, the number of connected nodes is more and the importance of nodes in the network is greater. Finally, we selected the top five compounds with the highest degree values for our follow-up study.

#### Protein–Protein Interaction Network

Common targets of “QJC-component-target-diarrhea” were structured using the online database Search Tool for the Retrieval of Interacting Genes/Proteins (STRING; https://string-db.org/) to predict the protein–protein interaction (PPI). The parameter organism was *S. scrofa* (pig), and the interaction scores were ≥0.9 (24) and hid disconnected nodes in the network.

#### Gene Ontology Analysis and Kyoto Encyclopedia of Genes and Genomes Pathways

The common genes of “QJC-component-target-diarrhea” were searched against the Database for Annotation, Visualization, and Integrated Discovery (DAVID; https://david.ncifcrf.gov/summary.jsp) and subjected to Gene Ontology (GO) and Kyoto Encyclopedia of Genes and Genomes (KEGG) pathway analyses. Species and background were limited to *S. scrofa* (pig). The downloaded results were sorted using false discovery rate (FDR) values and count values.

### Preparation of the Crude Extract and Fractions

HQ, SJ, and CQC were obtained from Harbin Chinese herbal market (Harbin, China). All herbal materials were identified by Professor Xiuju Wu (College of Life Sciences, Northeast Agricultural University, Harbin, China) and preserved in the laboratory of animal pharmacy, Northeast Agricultural University. The above herbal materials were crushed at room temperature using a leading-coming-air type crushing machine (You Qi machinery, FDV). Then, SJ, HQ, and CQC were accurately weighed (10,000 g each) with a precise analytical balance (Sartorius, GL124I-1SCN) and extracted with methanol (100.0 ml) by the maceration method for 8 h. The extract was subjected to ultrasonic extraction by an ultrasonic cleaner (KQ3200E) for 40 min to accelerate the dissolution of the target substance in methanol. The methanol extract was concentrated and evaporated to 10 ml by the rotary evaporator used (R3, BUCHI Labor Technik AG).

### High-Performance Liquid Chromatographic Analytical Conditions

The high-performance liquid chromatographic (HPLC) analysis of methanolic extract was performed using a Waters e2695 HPLC system (Shanghai Kezhe) equipped with a thermo C18 column (4.6 × 250 mm, 5 μm). All samples were filtered through a membrane of 0.45-μm before injection.

We used anhydrous methanol to prepare quercetin standard solutions with concentrations of 0.006, 0.012, 0.024, 0.048, 0.096, and 0.192 mg/ml and kaempferol standard solutions with concentrations of 0.00125, 0.0025, 0.005, 0.01, 0.02, and 0.04 mg/ml and establish calibration curves of quercetin and kaempferol. The quantitative determination conditions of quercetin and kaempferol were as follows: the mobile phase comprised a mixture of anhydrous methanol−0.2% phosphoric acid (65:35) with the flow rate of 1.0 ml/min, injection volume was 10 μl, the temperature of column was maintained at 30°C (25), and the detection was set at 360 nm (26).

Concentrations of 0.015625, 0.03125, 0.0625, 0.125, 0.25, and 0.5 mg/ml of the standard solution of stigmasterol were configured using anhydrous methanol to establish the calibration curve of stigmasterol. The mobile phase was anhydrous methanol with the flow rate of 1.0 ml/min, injection volume was 10 μl, the temperature of the column was maintained at 30°C, and the wavelength used was 208 nm; these conditions were used for the quantitative determination of stigmasterol.

We used anhydrous methanol to prepare scutellarein standard solutions with concentrations of 0.000391, 0.000781, 0.001563, 0.003125, 0.00625, and 0.0125 mg/ml and luteolin standard solutions with concentrations of 0.00625, 0.0125, 0.025, 0.05, 0.01, and 0.02 mg/ml and establish a calibration curve of scutellarein and luteolin. We used gradient elution with anhydrous methanol (solvent A) and 0.2% phosphoric acid (solvent B) as the mobile phase, and the injection volume was 10 μl. For scutellarein, the elution conditions were as follows: 0–3 min, 5–22% A; 3–15 min, 22–60% A; 15–20 min, 60–70% A; 20–30 min, 70–100% A; and 30–50 min, 100–5% A. For luteolin, the elution conditions were as follows: 0–8 min, 55–62% A; 8–15 min, 62–70% A; and 15–35 min, 70–55% A. The UV detection wavelength was set at 335 and 350 nm, respectively. The flow rate was 0.6 ml/min, and the column temperature was maintained at 30°C (8).

For quantitative analysis, the calibration curve of quercetin, luteolin, kaempferol, stigmasterol, and scutellarein was constructed by plotting the peak area vs. concentration curve; at the same time, the regression equations were calculated. Then, the regression equations were used to calculate the content of each component in the sample. All samples were analyzed in triplicate.

### Animals

#### Chemicals and Reagents

Quercetin (No. T2174, purity = 97.27%), luteolin (No. T1027, purity = 96.61%), kaempferol (No. T2177, purity = 99.41%), stigmasterol (No. T2967, purity = 99.84%), and scutellarein (No. T3319, purity = 99.32%) were purchased from Topscience (Shanghai, China) and kept in −80°C. Both loperamide hydrochloride (No. B33838) and serotonin hydrochloride (B21833) were obtained from Shanghai Yuanye (Shanghai, China) and stored at 4°C. Sodium carboxymethyl cellulose (CMC) (No. MB1731) was provided by Meilunbio (Dalian, China). Fast Start DNA Master SYBR Green (Roche, Basel, Switzerland) was stored in the dark at −20°C. The RNA extraction kit (DP431) was kept in 20°C, and the reverse transcription kit (KR118) was stored at −20°C; both were purchased from Tiangen (Beijing, China).

#### Experimental Procedures

A total of 85 Kunming male mice (age, 6–8 weeks) were purchased from the experimental animal center of Harbin Medical University. The animals were housed at the animal house of the Northeast Agricultural University. They were given adequate water and provided standard diet. All procedures used in this experiment were approved by the Institutional Animal Care and Use Committee of Northeast Agricultural University (No. NEAUEC20). Welfare-related assessments and interventions were carried out before, during, or after establishing the mouse model for experiments. Ten mice were fasted for 18 h before the experiment, and water was made available *ad libitum*. Then, the mice were divided into normal control (NC) group and model control (MC) group. The mice of the MC group were intraperitoneally injected with 0.5 ml of serotonin hydrochloride (dissolved in normal saline) at a concentration of 5 mg/kg, and the mice of the NC group received intraperitoneal injection of 0.5 ml saline (27). The animals were placed in cages lined with white paper and observed for 3 h, and the rates of loose stools were calculated according to the accumulated number of stools and the number of loose stools in mice to evaluate whether the mouse diarrhea model was successfully established.


$$\begin{array}{l} \text{ The rates of loose stools }\left(\%\right)\\ \text{=}\frac{\text{The number of loose stools}}{\text{The accumulated number of stools}}\times 100\% \end{array}$$

#### Determine the Best Therapeutic Dose

Thirty mice were fasted for 18 h before the experiment, and water remained available for consumption *ad libitum*. We randomly divided mice into six groups, with five mice per group; the groups were NC group, MC group, and positive control (PC) group, and different dose groups, including high-dose (HD) group, mid-dose (MD) group, and low-dose (LD) group. Normal control and MC groups were orally administered 0.2 ml of 5% sodium CMC by intragastric gavage. The PC group was administered loperamide hydrochloride (0.5 mg/kg) by intragastric gavage. The remaining three groups were given QJC 30, 60, and 120 mg/kg as low dose, mid dose, and high dose, respectively. Except for mice in the NC group, diarrhea was induced in each group by 5-mg/kg serotonin hydrochloride intraperitoneal injection 30 min after administration (27). The time of initial diarrhea was measured, and the accumulated number of stools, the number of loose stools, and the rates of loose stools within 6 h were measured.

#### The Effect of Active Ingredients of QJC on Diarrhea

Here, 45 mice were fasted for 18 h before the experiment, and water remained available for consumption *ad libitum*. Then, the mice were randomly divided into nine groups with five mice per group. Normal control, MC, and PC groups were the same as mentioned previously. The mice of drug treatment groups received intragastric administration of QJC (30 mg/kg), quercetin (50 mg/kg), luteolin (5 mg/kg), kaempferol (5 mg/kg), stigmasterol (200 mg/kg), and scutellarein (1.75 mg/kg). All these drugs were dissolved in CMC. Except the NC group, the diarrhea model was established by intraperitoneal injection of 5-mg/kg serotonin hydrochloride (dissolved in normal saline) 30 min after administration (27). After the model was made, the time of initial diarrhea, the accumulated number of stools, the number of loose stools, and the rates of loose stools of mice within 6 h were measured.

#### Histologic Examination

The duodenum, jejunum, and ileum of each mouse were taken and redundant tissues were carefully pruned, waste was eliminated, and kept in a 50-ml centrifuge tube filled with 4% formaldehyde solution. Then, dehydration and paraffin embedding were performed, and 5-mm sections were cut and stained with hematoxylin and eosin (H&E). Morphologic changes in the intestinal villi, crypt depth, and goblet cells were observed under a microscope (28). ImageJ was used (version 1.52) to measure villus height, crypt depth, and villus height/crypt depth. Goblet cells were counted.

### Quantitative Real-Time PCR Analysis

Total RNA from the duodenum, jejunum, and ileum was extracted using an RNAprep Pure Tissue Kit (DP431, Tiangen Biotech, Beijing). The RNA concentration and purity were determined by spectrophotometry (NanoDrop 2000, Thermo, USA). The *phosphoinositide 3-kinase* (*PI3K*), *Akt*, and *phosphatase and tensin homolog* (*PTEN*) primers were designed by Primer5.0, synthesized by COME (Jilin, China), and stored at −80°C for real-time PCR. The experiment was performed under the guidance of the instruction manual. The expression levels of *PI3K, Akt*, and *PTEN* were quantified relative to the expression of β*-actin* as the endogenous control by the 2^−ΔΔCt^ method (29–31). Primer sequences are shown in Table 1.

**Table 1:** Primers used for the RT-qPCR analysis.

**Target gene**	**Primer sequence**	**Product length**	**Amplification efficiencies (%)**
*PI3K*	F: 5′-GCCCCGGGTAGGTCTAGATT-3′	104	96.65
	R: 5′-CATGCCCTATGCGACCTGA-3′		
*Akt*	F: 5′-CCCTGCTCCTAGTCCACCA-3′	85	98.61
	R: 5′-TGTCTCTGTTTCAGTGGGCTC-3′		
*PTEN*	F: 5′-CAGCCATCATCAAAGAGATCG-3′	113	101.83
	R: 5′-TGCAGGAAATCCCATAGCAA-3′		
β-*actin*	F: 5′-TGACGTGGACATCCGCAAAG-3′	205	98.37
	R: 5′-CTGGAAGGTGGACAGCGAGG-3′		

*F, forward; R, reverse; PI3K, phosphoinositide 3-kinase; PTEN, phosphatase and tensin homolog*.

### Statistical Analysis

Statistical analysis was performed with the SPSS software (version 17.0), and the results were expressed as mean ± standard deviation (SD). The Student's *t*-test or one-way analysis of variance (ANOVA) was performed to analyze group differences, and *p* < 0.05 was considered an indicator of a statistically significant difference. Moreover, the pictures were plotted using GraphPad Prism 5.0 software.

## Results

### Active Compounds in QJC

Here, 35 components were identified in QJC. It is worth noting that stigmasterol is a common component of SJ and CQC. The 35 bioactive compounds are displayed in Supplementary Table 1. At length, 446 gene targets of QJC were obtained.

### Identifying the Common Targets

**Figure 2:**
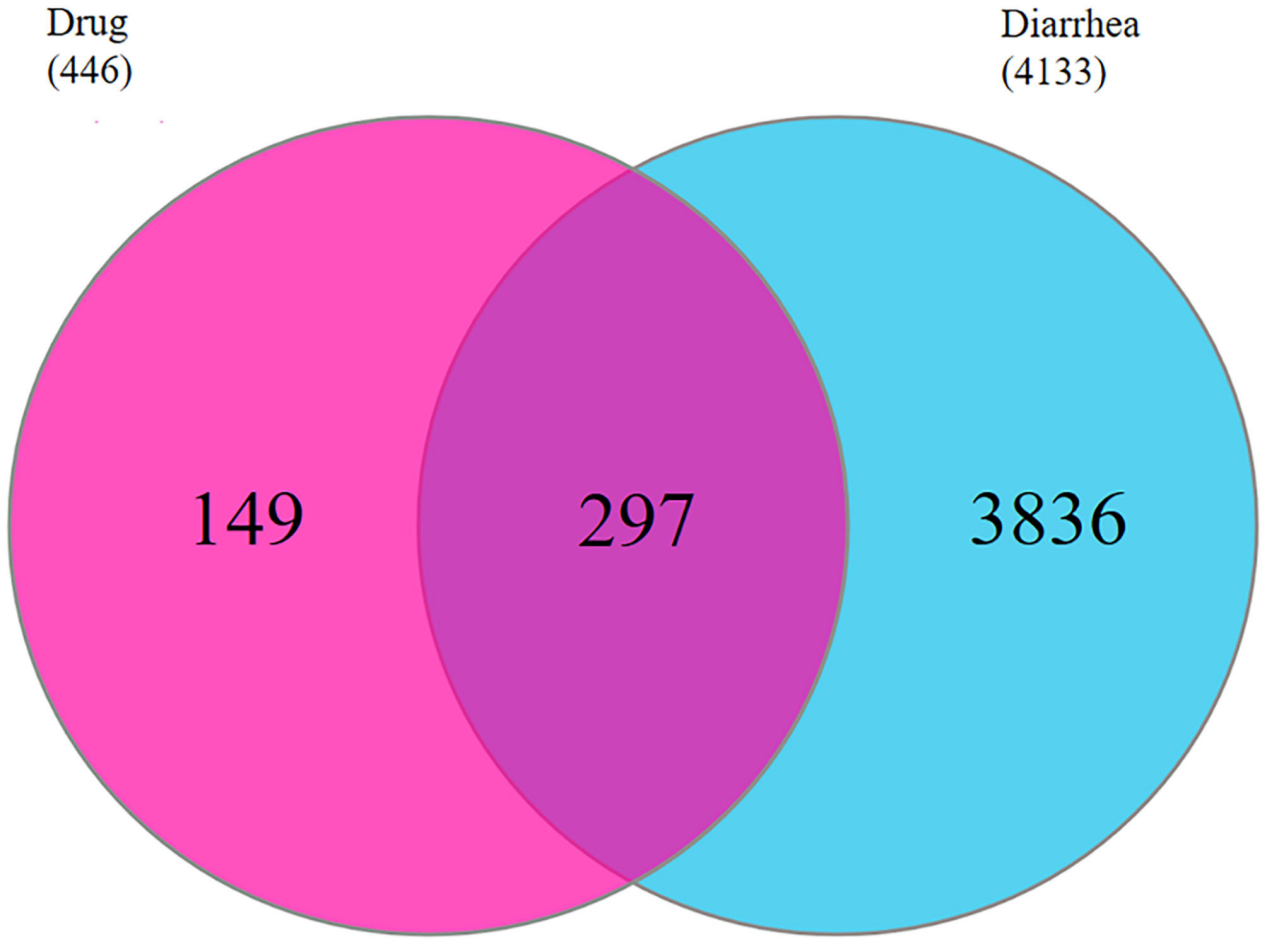
The common targets of QJC and diarrhea were identified. The red circle represents 446 targets of QJC; the blue circle represents 441 targets of diarrhea; and the purple circle represents 297 common targets.

**Figure 3:**
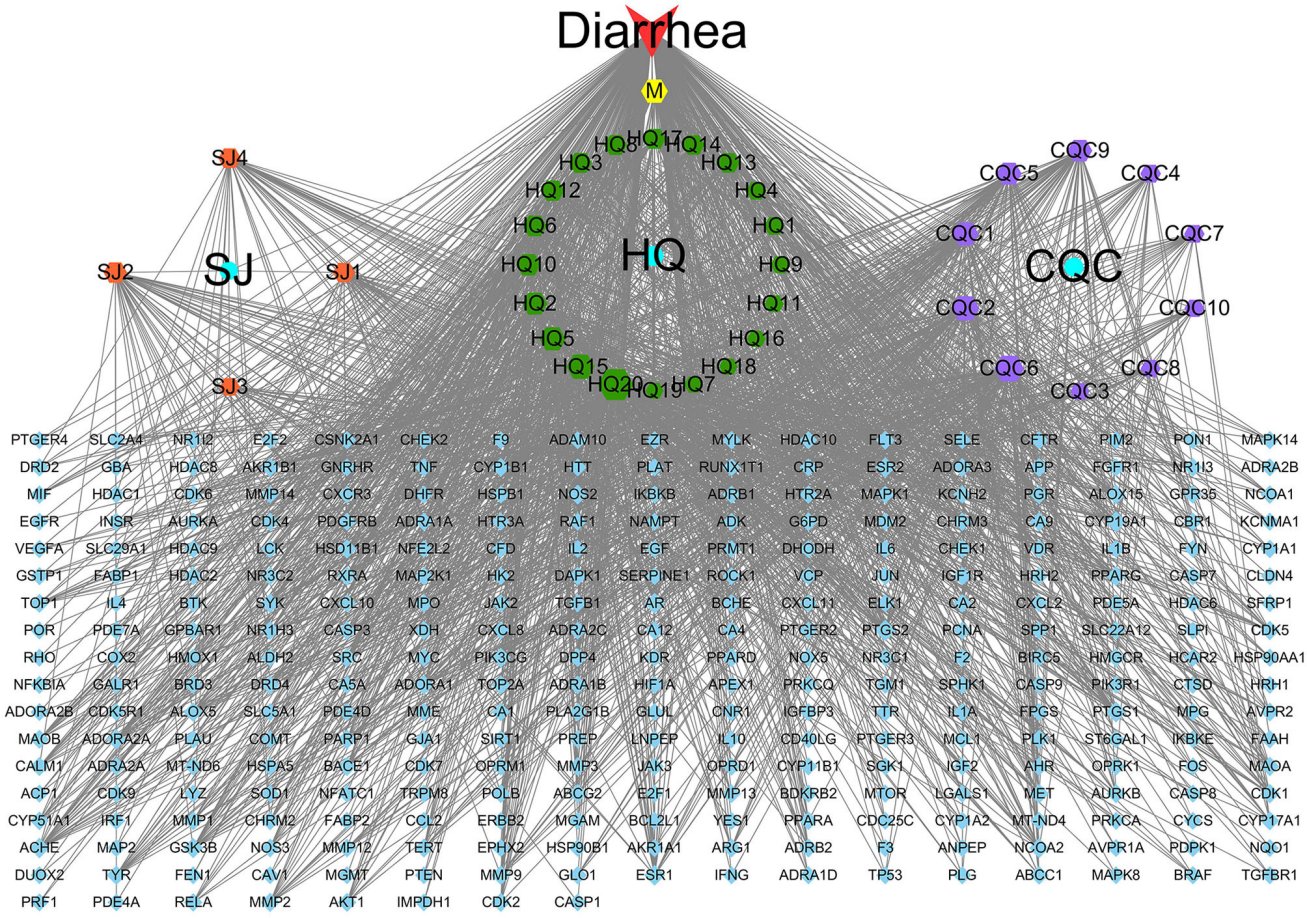
The “QJC-component-target-diarrhea” network. The yellow hexagon represents stigmasterol; the blue circle represents *Zingiber officinale* Roscoe (SJ), *Astragalus propinquus* Schischkin (HQ), and *Plantago asiatica* L. (CQC); the blue parallelogram represents overlapping genes; the orange parallelogram represents components of SJ; the green hexagon represents components of HQ; and the purple hexagon represents components of CQC. The node size represents the size of the degree.

Here, 4,133 targets were obtained. Subsequently, 297 overlap genes were identified by employing Interactive Venn online tools (Figure 2). Next, the common targets were used to construct a “QJC-component-target-diarrhea” network (Figure 3), which had 336 nodes and 1,832 edges by the Cytoscape 3.7.2 software. The network showed that the efficacy of QJC in treating diarrhea was based on the synergistic effect of different components on multiple targets. Moreover, depending on their degree in the network, the top 10 active constituents were quercetin (degree = 148, HQ20), luteolin (degree = 98, CQC6), kaempferol (degree = 86, HQ15), baicalein (degree = 83, CQC2), stigmasterol (degree = 76, M), isorhamnetin (degree = 72, HQ5), dinatin (degree = 71, CQC1), jaranol (degree = 62, HQ2), 6-OH-luteolin (degree = 62, CQC5), and scutellarein (degree = 59, CQC9). Finally, we chose quercetin, luteolin, kaempferol, scutellarein, and stigmasterol to treat stress diarrhea in mice.

### Protein–Protein Interaction Network Analysis

**Figure 4:**
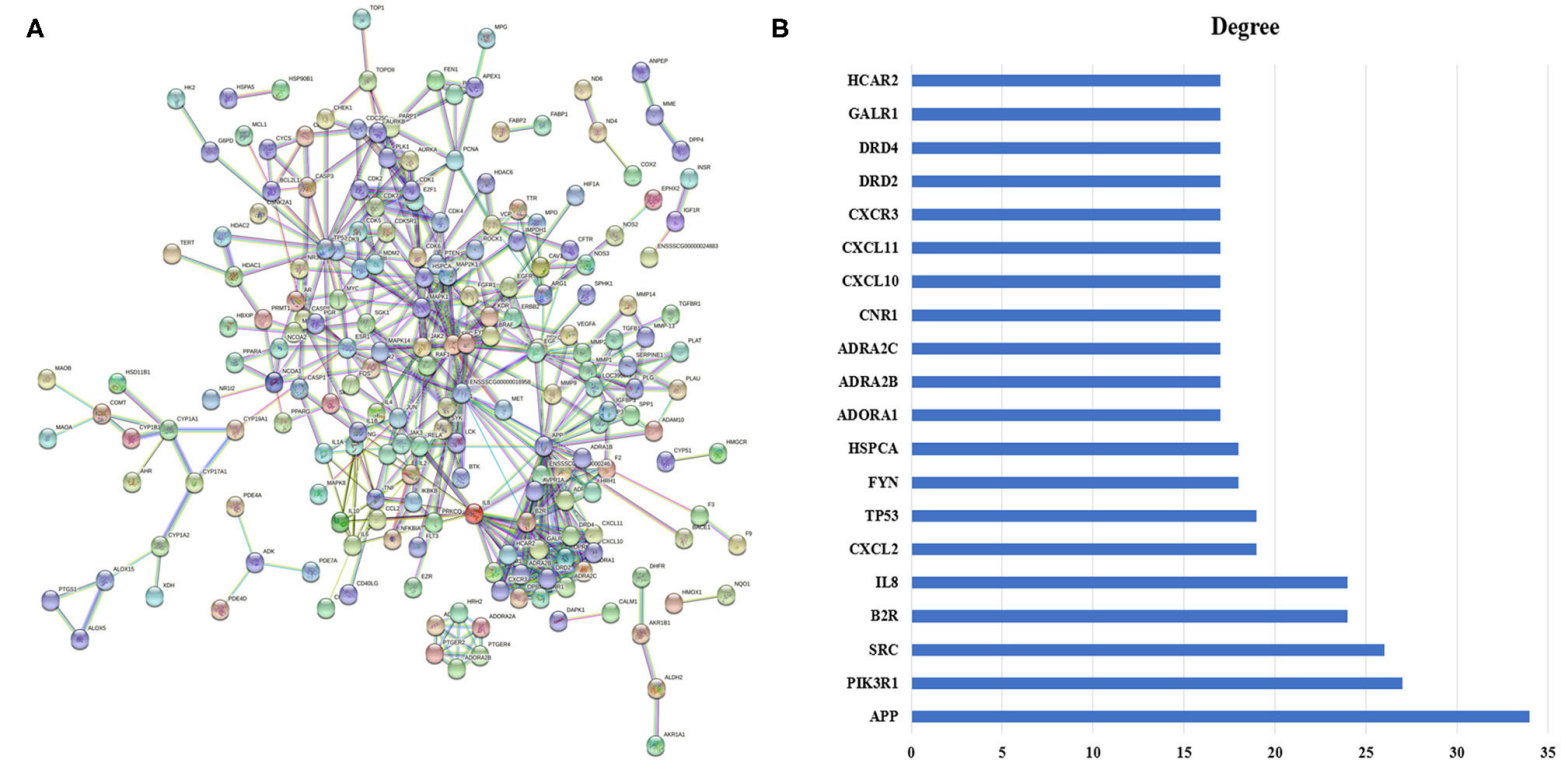
Protein–protein interaction (PPI) network and its top 20 genes. **(A)** PPI network: The node represents the relevant gene, and the line width of the edge indicates the strength of the data support. **(B)** The top 20 genes of the PPI network: X-axis represents degree, and Y-axis indicates genes.

The PPI network comprised 203 nodes and 595 edges (Figure 4A), which indicated 595 interactions. To clarify this further, the top 20 nodes were extracted by the degree (Figure 4B). In the interaction network, amyloid beta A4 protein (APP; degree = 34), phosphoinositide-3-kinase regulatory subunit 1 (PIK3R1; degree = 27), Src protein-tyrosine kinase (SRC; degree = 26), B2 bradykinin receptor (B2R; degree = 24), and IL-8 (degree = 24) are the top five potential targets of QJC in the treatment of diarrhea, which may play an important role.

### Gene Ontology and Kyoto Encyclopedia of Genes and Genomes Enrichment Analyses

**Figure 5:**
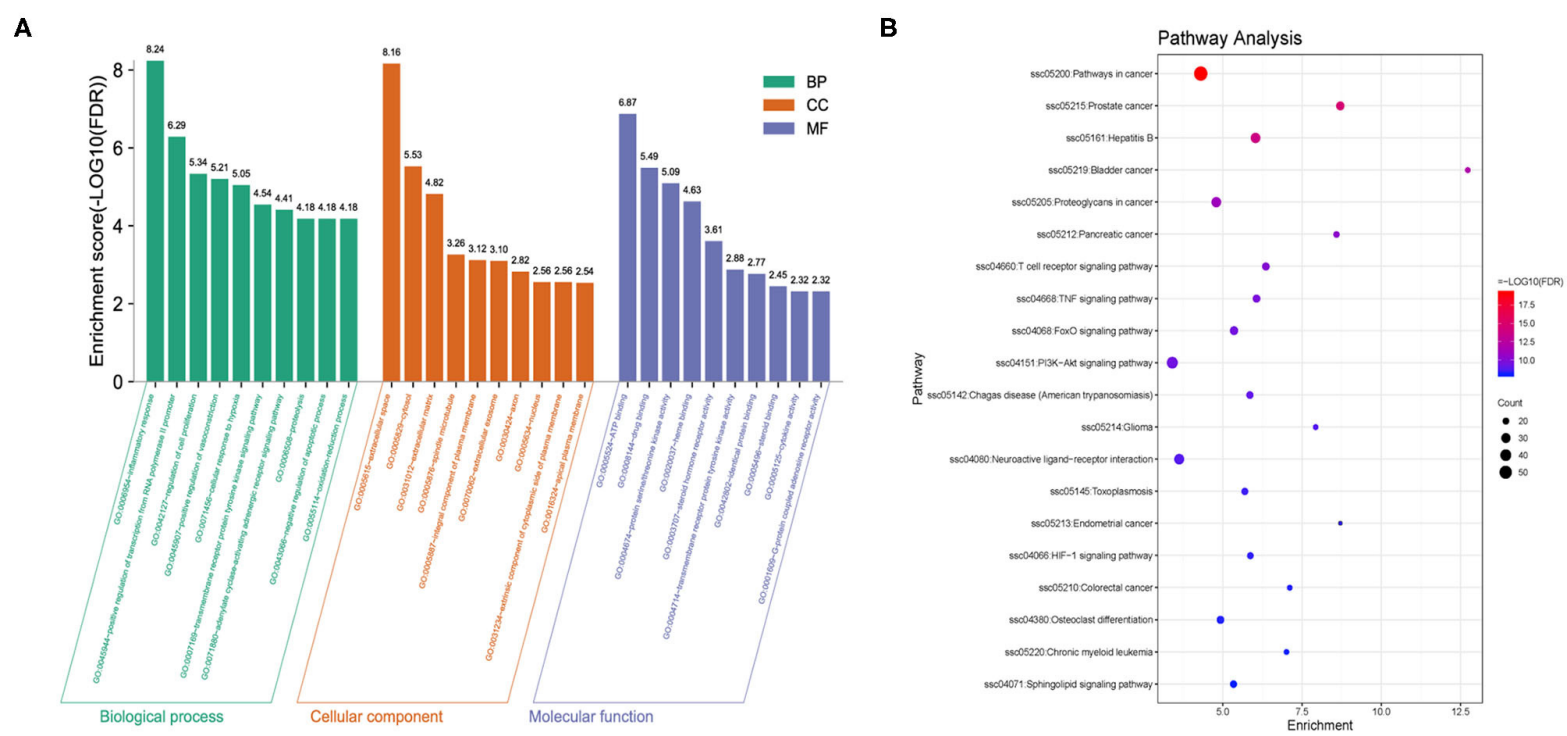
Gene Ontology (GO) and Kyoto Encyclopedia of Genes and Genomes (KEGG) analyses of targets. **(A)** GO analysis of targets: X-axis represents GO terms, and Y-axis indicates the enrichment score [adjusted false discovery rate (FDR)] in that term. **(B)** KEGG analysis of targets: Dot plot showed the top 20 KEGG pathways with adjusted FDR. The color scale indicates the adjusted FDR, and the dot size represents the gene count in each term.

To further explore the biological characteristics of the 297 candidate targets of QJC, we performed GO analysis on diarrhea targets. Consequently, the biological process (BP) was involved in 292 enrichment results, the molecular function (MF) was involved in 44 enrichment results, and the cellular component (CC) was involved in 73 enrichment results. Based on their enrichment score, we visualized the top 10 pathways (Figure 5A).

Kyoto Encyclopedia of Genes and Genomes (KEGG) pathway analysis was used to explore the potential mechanisms of QJC in the treatment of stress diarrhea. Consequently, 130 KEGG pathways were obtained, and the top 20 KEGG signaling pathways were constructed based on FDR (Figure 5B).

### High-Performance Liquid Chromatographic Results

Quercetin, kaempferol, luteolin, scutellarein, and stigmasterol were eluted, as shown in Supplementary Figure 1. Meanwhile, we calculated the content of different components in the sample using the regression equation. Quercetin, kaempferol, luteolin, scutellarein, and stigmasterol levels were 4.91 ± 0.228, 2.18 ± 0.237, 25.58 ± 0.543, 0.53 ± 0.026, and 145.72 ± 13.712 μg/g, respectively (Supplementary Table 2).

### Anti-diarrhea Effect of QJC and Its Active Ingredients

**Figure 6:**
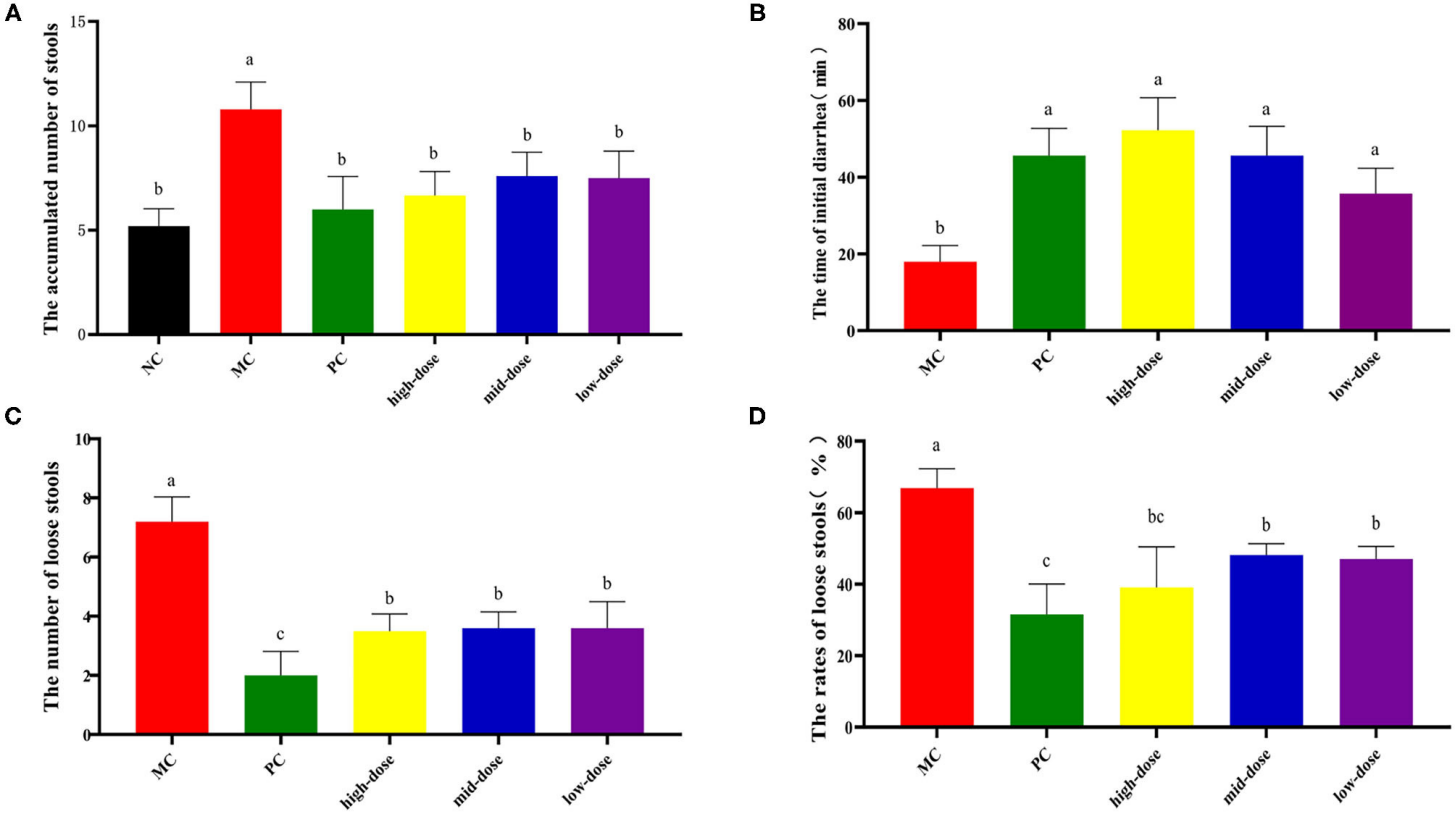
Treatment of diarrhea mice with gradient dose of QJC. Data were expressed as the mean ± SD (*n* = 5); the significant difference between groups (*p* < 0.05) was shown by the different letters above the histogram. **(A)** The accumulated number of stools. **(B)** The time of initial diarrhea. **(C)** The number of loose stools. **(D)** The rates of loose stools.

Our preliminary experimental results showed that compared with the NC group, the cumulative defecation times of the model group mice increased significantly (*p* < 0.05), indicating that the diarrhea model was successfully established (Table 2). After receiving 30-, 60-, and 120-mg/kg QJC, the accumulated number of stools, time of initial diarrhea, number of loose stools, rates of loose stools of mice in the high-dose, mid-dose, and low-dose groups were significantly different from those in the MC group (*p* < 0.05), and no dose dependence was noted (Figure 6).

**Table 2:** Establishment of a mouse model of stress diarrhea.

**Group**	**The accumulated number of stools**	**The number of loose stools**	**The rates of loose stools (%)**
Normal control group	3.8 ± 0.838^a^	0^a^	0^a^
Model control group	11.6 ± 1.141^b^	7.8 ± 0.837^b^	0.68 ± 0.074^b^

a,b *Significant differences at p < 0.05*.

**Figure 7:**
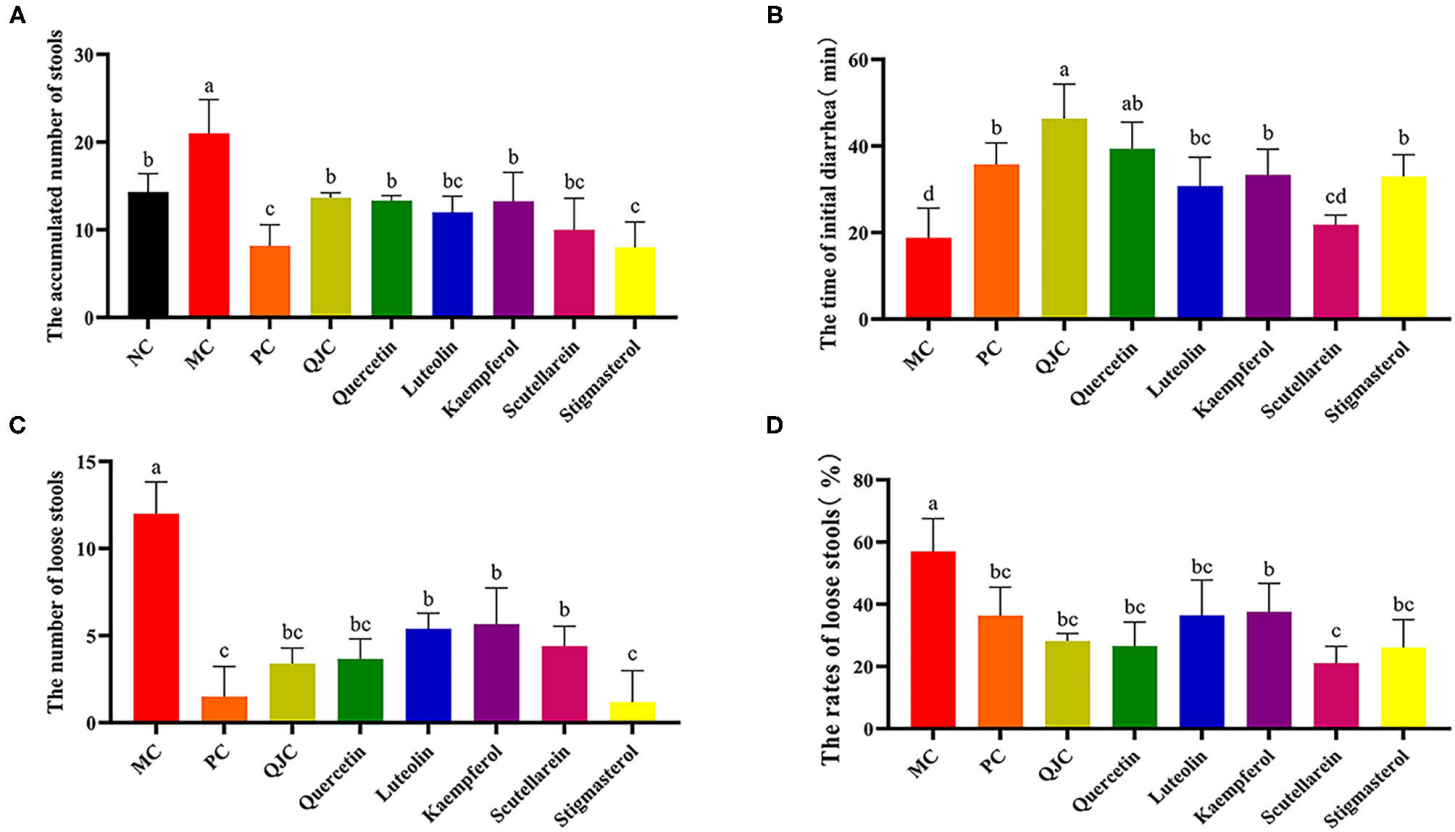
Treatment of diarrhea mice with QJC and its five active ingredients. Data were expressed as the mean ± SD (*n* = 5); the significant difference between groups (*p* < 0.05) was shown by the different letters above the histogram. **(A)** The accumulated number of stools. **(B)** The time of initial diarrhea. **(C)** The number of loose stools. **(D)** The rates of loose stools.

The diarrhea symptoms resolved to varying degrees after treatment with QJC, quercetin, kaempferol, luteolin, scutellarein, and stigmasterol (Figure 7). These results suggested that QJC could effectively exert anti-diarrhea activity (*p* < 0.05).

### Histomorphology of Intestinal Mucosa

**Figure 8:**
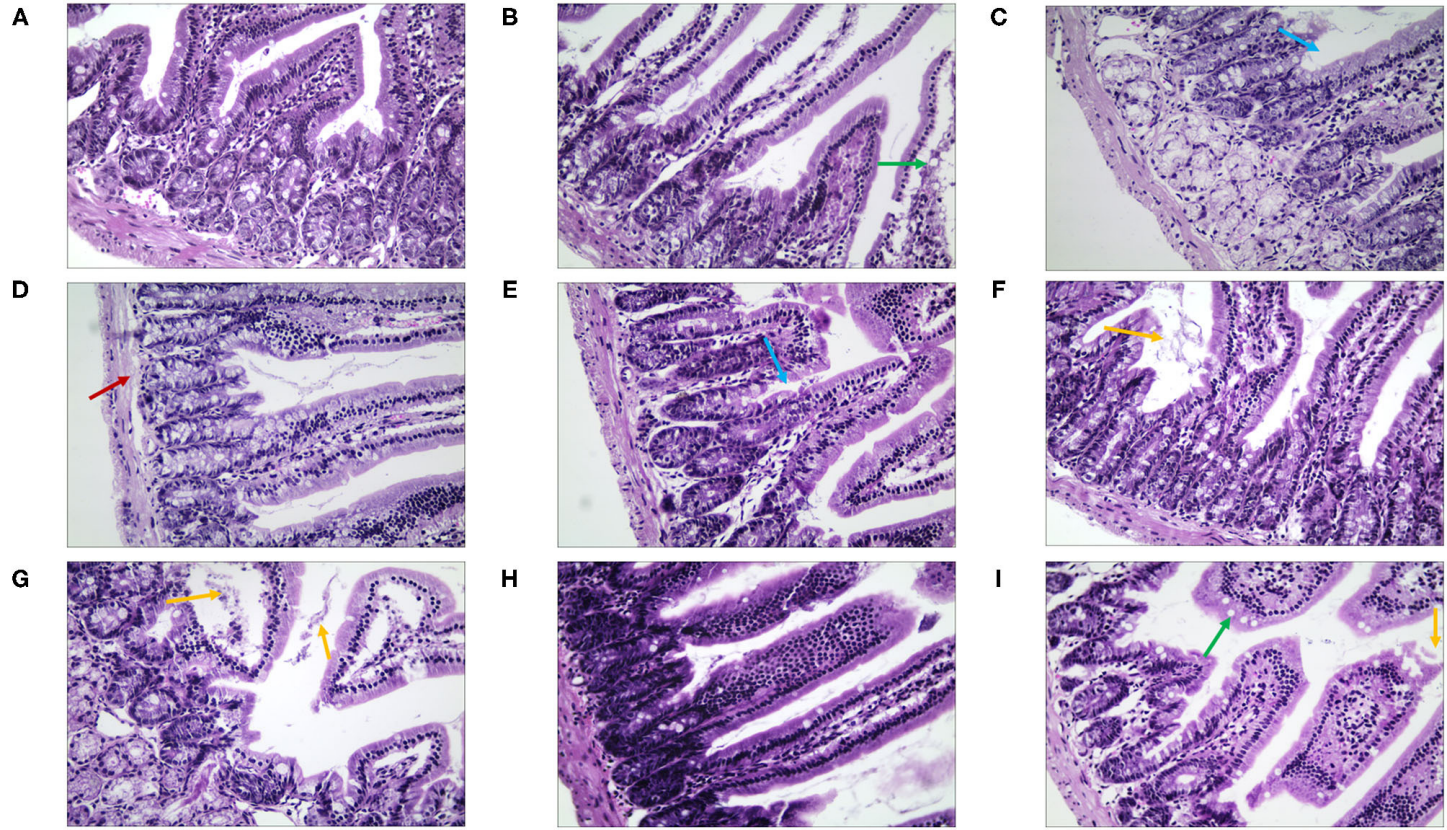
The morphological and histopathological changes (H&E, ×50 magnification) of the duodenum tissues in diarrhea mice. **(A)** Normal control (NC) group, **(B)** model control (MC) group, **(C)** positive control (PC) group, **(D)** QJC group, **(E)** quercetin group, **(F)** kaempferol group, **(G)** luteolin group, **(H)** scutellarein group, and **(I)** stigmasterol group. Yellow arrow: exfoliated mucosal epithelial cells; blue arrow: intestinal crypts; green arrow: goblet cells; red arrow: loose connective tissue; *n* = 5 per group.

We observed morphological and histopathological changes in the duodenum, jejunum, and ileum in mice with stress diarrhea. In the duodenum, we found in the NC group that the structure of the intestinal tissue was clear, and the epithelial cells were high columnar cells (Figure 8). Compared with the NC group, in the MC group, duodenal injury was serious, intestinal tissue exhibited evident edema, the goblet cells were fewer, and the intestinal villi were shorter. The structure of the duodenal tissue of the QJC group was completely clear. In quercetin and kaempferol groups, mucosal structural integrity was maintained; but villus was slightly edematous. However, luteolin, scutellarein, and stigmasterol groups showed more exfoliated mucosal epithelial cells, and the intestinal villi of the scutellarein group were also shorter (Supplementary Table 3).

**Figure 9:**
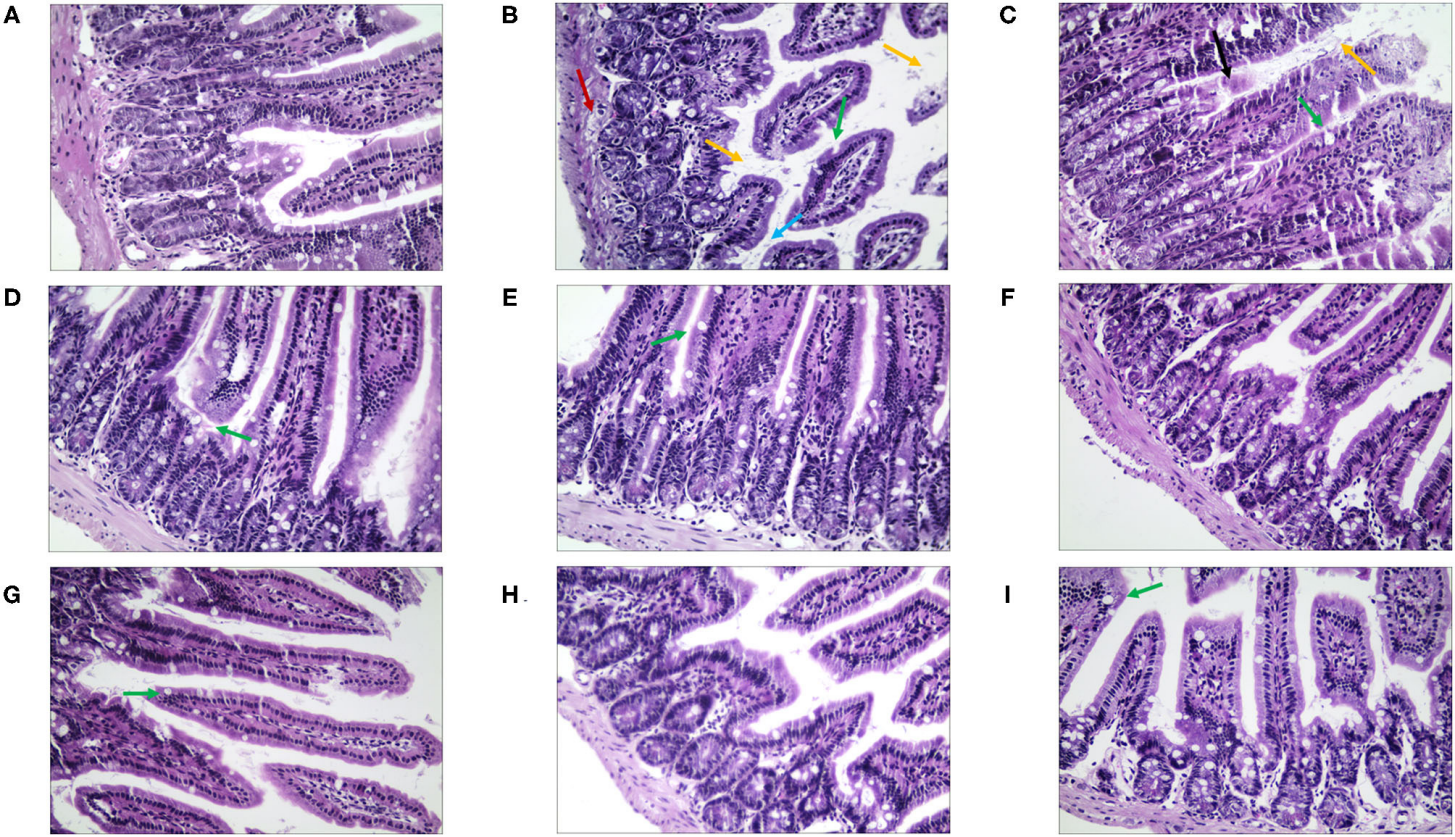
The morphological and histopathological changes (H&E, ×50 magnification) of the jejunum tissues in diarrhea mice. **(A)** Normal control (NC) group, **(B)** model control (MC) group, **(C)** positive control (PC) group, **(D)** QJC group, **(E)** quercetin group, **(F)** kaempferol group, **(G)** luteolin group, **(H)** scutellarein group, and **(I)** stigmasterol group. Yellow arrow: exfoliated mucosal epithelial cells; blue arrow: intestinal crypts; green arrow: goblet cells; red arrow: loose connective tissue; original magnification: ×50, *n* = 5 per group.

Then, in terms of the pathological changes in the jejunum (Figure 9), compared with the NC group, the mice of the MC group showed serious edema, the mucosal epithelial cells were shedding, the crypts were deeper, and the goblet cell count was lower (Supplementary Table 4). The QJC group had dense jejunal villi and no evident edema. scutellarein and stigmasterol groups showed that the structure of the jejunal lining was continuous and without deformities. Kaempferol and luteolin groups showed slight shedding of jejunal epithelial cells; however, the number of goblet cells and the parameters of villus height/crypt depth were better than those in the MC group.

**Figure 10:**
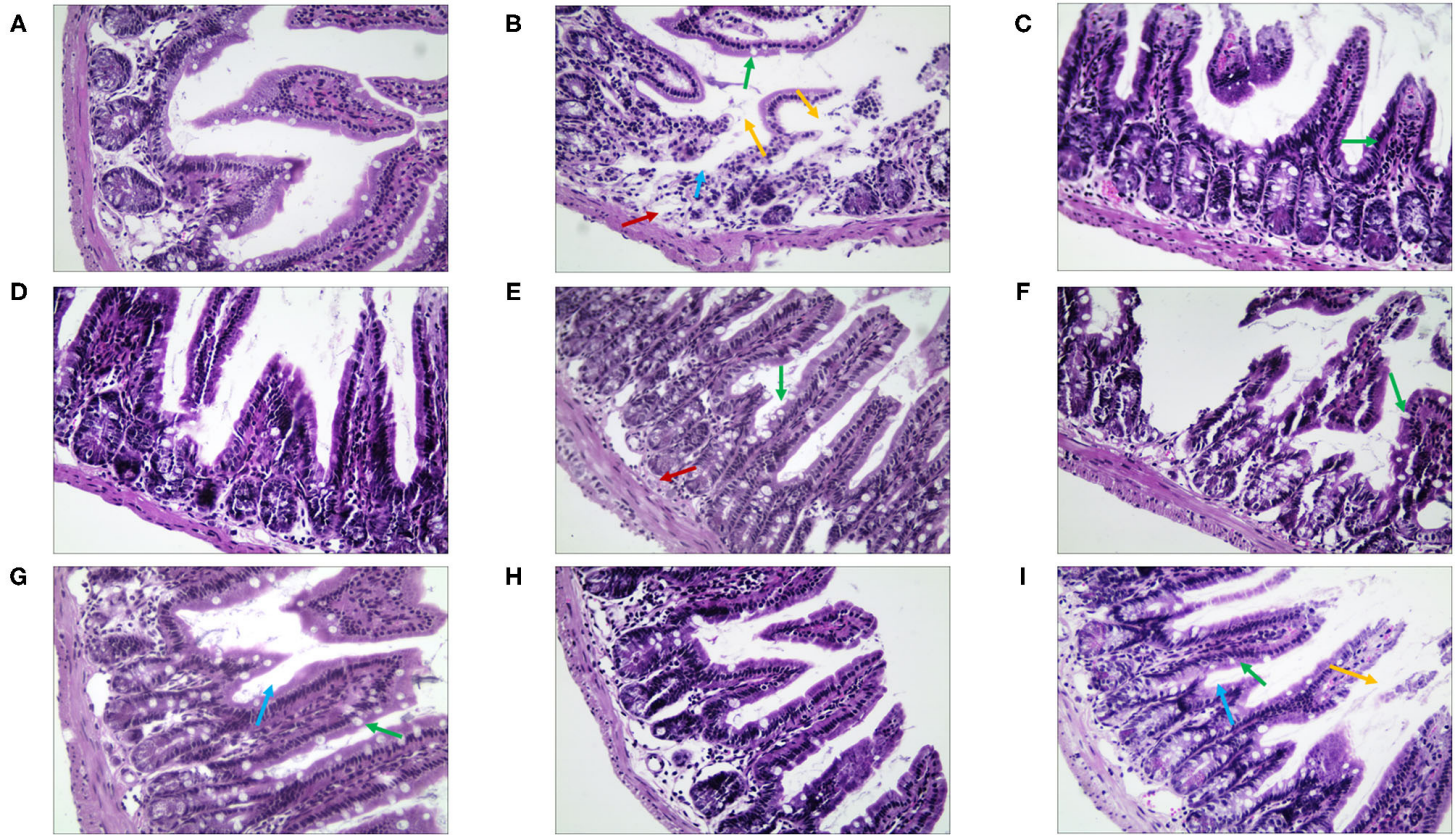
The morphological and histopathological changes (H&E, ×50 magnification) of the ileum tissues in diarrhea mice. **(A)** Normal control (NC) group, **(B)** model control (MC) group, **(C)** positive control (PC) group, **(D)** QJC group, **(E)** quercetin group, **(F)** kaempferol group, **(G)** luteolin group, **(H)** scutellarein group, and **(I)** stigmasterol group. Yellow arrow: exfoliated mucosal epithelial cells; blue arrow: intestinal crypts; green arrow: goblet cells; red arrow: loose connective tissue; original magnification: ×50, *n* = 5 per group.

By observing the pathological changes in the ileum (Figure 10), we found that there were numerous goblet cells in the ileum in the NC group. The mice of the MC group showed severe edema, highly disordered ileal structure, mucosal structural disorder, dramatically reduced goblet cell count, and fewer local crypts (Supplementary Table 5). However, there was significant improvement in the treatment group. The villi structure of the QJC group was clear and complete, but still, a few epithelial cells appeared exfoliated. Quercetin, luteolin, and stigmasterol groups could evidently depress the abscission of epithelial cells of the ileal mucosa and showed that the number of goblet cells and the parameters of villus height/crypt depth were better compared with those of the MC group.

### Effects of QJC on the PI3K–Akt Signaling Pathway in Serotonin Hydrochloride-Infected Mice

**Figure 11:**
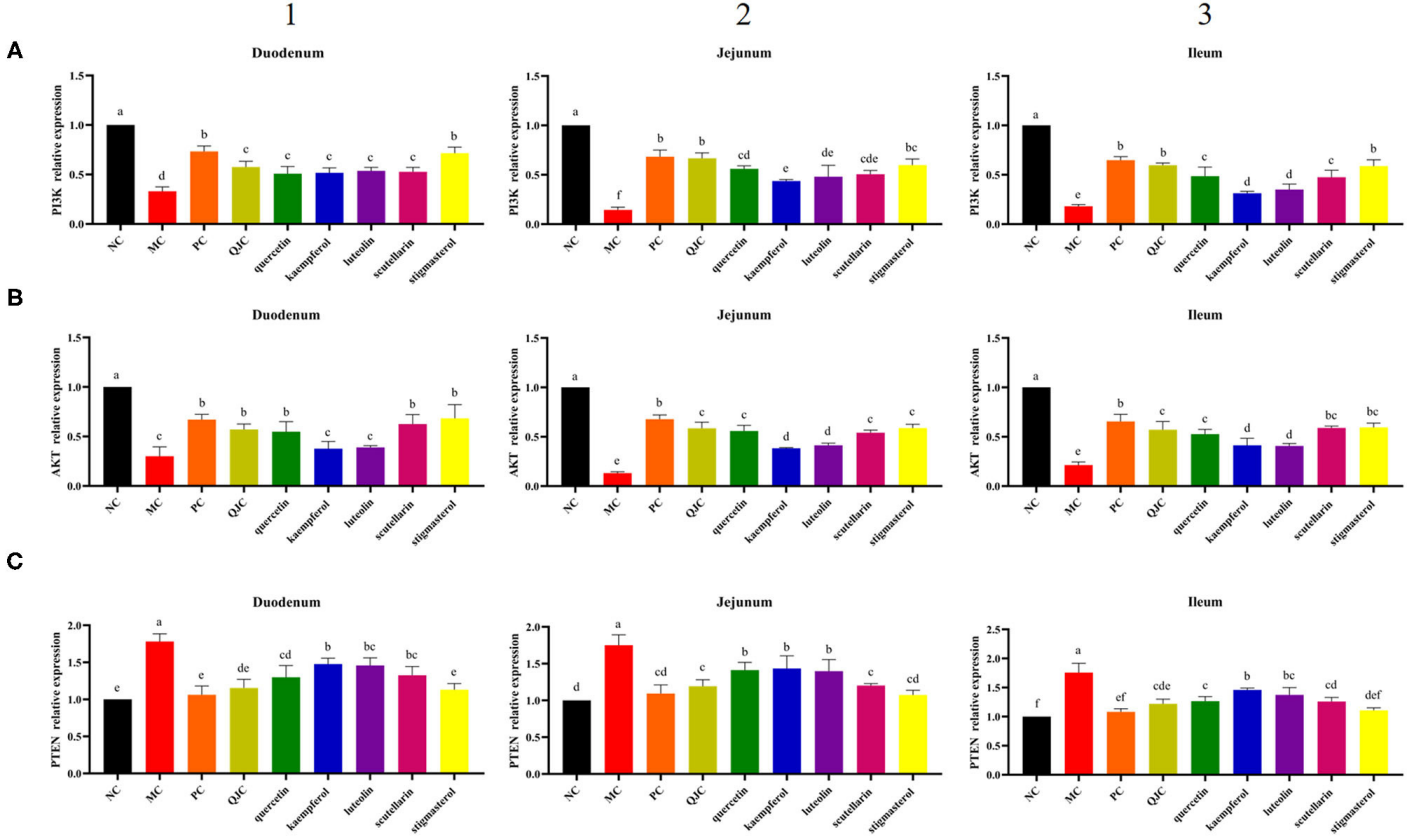
Effect of QJC and its five active ingredients on the mRNA expression in diarrhea mouse small intestine tissues. The significant difference between groups (*p* < 0.05) was shown by the different letters above the histogram. **(A)** The *phosphoinositide 3-kinase* (*PI3K*) relative expression in the duodenum, jejunum, and ileum. **(B)** The *Akt* relative expression in the duodenum, jejunum, and ileum. **(C)** The *phosphatase and tensin homolog* (*PTEN*) relative expression in the duodenum, jejunum, and ileum. **(1)** The *PI3K, Akt*, and *PTEN* relative expression in the duodenum. **(2)** The *PI3K, Akt*, and *PTEN* relative expression in the jejunum. **(3)** The *PI3K, Akt*, and *PTEN* relative expression in the ileum.

Quantitative real-time PCR (RT-qPCR) results (Figure 11) showed that in the duodenum, jejunum, and ileum, compared with the NC group, the MC group relative expression levels of *PI3K* and *Akt* were significantly decreased (*p* < 0.05), and the relative expression levels of *PTEN* were significantly increased (*p* < 0.05). Moreover, compared with the MC group, the expression levels of *PI3K* and *Akt* were increased in each drug treatment group (*p* < 0.05), whereas the expression levels of *PTEN* were decreased (*p* < 0.05). However, these indices mentioned above could not be completely restored to normal levels in the drug treatment groups. Meanwhile, we found that the therapeutic effect of QJC and stigmasterol to treat stress diarrhea may be consistent with loperamide hydrochloride (*p* > 0.05).

## Discussion

Notably, TCM has complex components, making it difficult to elucidate its specific mechanisms. Simultaneously, both network pharmacology and TCM have the characteristics of being multi-component, multi-target, and multi-pathway approaches and are widely implemented to investigate the correlation among biological systems, drugs, and diseases (32). Weaning is one of the crux stages in pig production; the period is usually accompanied by diarrhea, which incurs great economic losses (2). Preliminary clinical studies in our laboratory have shown that QJC, which is composed of *A. propinquus* Schischkin (HQ), *Z. officinale* Roscoe (SJ), and *P. asiatica* L. (CQC), has an effective anti-diarrhetic effect *in vivo*. In this study, the chemical components of QJC in the treatment of diarrhea were studied through the ADME analysis and topological analysis to predict the core compounds responsible for treating diarrhea. The results revealed quercetin, luteolin, kaempferol, scutellarein, and isorhamnetin as the main active compounds. Previous studies have reported that quercetin has anti-diarrhea effects (33) and that the underlying anti-diarrhea mechanism is associated with enhancing the epithelial barrier (34). Our previous research has revealed luteolin and scutellarein as the main anti-diarrhea components of CQC. They could increase the content of Na^+^ and K^+^ by upregulating the activity and gene level of creatine kinase and Na^+^/K^+^-ATPase, thereby helping alleviate diarrhea (8).

It is of great significance to integrate network pharmacology, which is based on big data bioinformatics, to uncover the molecular mechanisms of action of Chinese medicines. Kyoto Encyclopedia of Genes and Genomes enrichment results indicated that multiple signaling pathways may be involved in the anti-diarrhea effect. Bladder cancer, pancreatic cancer, the TNF signaling pathway, and the PI3K–Akt signaling pathway are all reportedly related to diarrhea. Current studies have shown that anti-TNF drugs can be used to exert anti-inflammatory effects, thereby effectively treating inflammatory bowel disease (35, 36); however, few studies have proven that drugs act directly through TNF signaling pathways. Conversely, the PI3K–Akt signaling pathway is directly related to stress-induced UC and irritable bowel syndrome (IBS) (37, 38). The PI3K–Akt signaling pathway is reportedly related to various diverse physiological stresses. It plays a crucial regulatory role during cellular stress (39–41) and is reportedly associated with stress-induced diarrhea, predominantly IBS (IBS-D), and UC (38, 42–44). In addition, the results of KEGG enrichment analysis show that the PI3K–Akt signaling pathway (number of enriched genes = 39) can enrich more genes than the TNF signaling pathway (number of enriched genes = 23). Thus, we assume that the effect of QJC in the treatment of diarrhea is related to the PI3K–Akt signaling pathway.

Recent research has found that 5-hydroxytryptamine (5-HT) in the gastrointestinal tract plays an important role in regulating growth and the maintenance of mucosa. Dong et al. (40) found that diarrhea in mice under weaning stress is accompanied by a significant increase in 5-HT content in the small intestine; besides this, citalopram hydrobromide can increase the secretion of 5-HT in mice and cause diarrhea. Therefore, we established a diarrhea model by intraperitoneal injection of serotonin hydrochloride to simulate weaning stress to study the pathological changes in the small intestine tissue of mice after QJC treatment. According to reports, weaning stress not only can lead to small intestine injury, decrease the height of villi, and change their morphology, such as changing from dense finger-like villi to smooth tongue-like villi, but also may also weaken the active absorption capacity of the small intestine (45). In our experiments, the duodenum, jejunum, and ileum of the MC group had varying degrees of damage. Among them, the goblet cells located on the mucosal surface could produce mucus, which is the main barrier to prevent microorganisms from infecting the healthy intestine (46). After treatment with drugs, the number of goblet cells slightly increased. Besides, the present study indicated that luteolin can prevent intestinal damage by attenuating villi shortening, vacuolization, and apoptosis and preserving the production of mucin in the duodenum and colon (47). Our work suggested that quercetin, kaempferol, scutellarein, and stigmasterol can alleviate the pathological condition of small intestine tissue and improve the integrity of the intestinal barrier.

Studies have shown that stress can cause UC. It is generally believed that UC is caused by imbalances in the expression of the molecules involved in pro-inflammatory and anti-inflammatory processes. At the same time, multiple pathways are associated with UC development, including the PI3K–Akt signaling pathway, mitogen-activated protein kinase (MAPK) signaling pathway, and nuclear factor (NF)-κB signaling pathway (48). In our study, the PI3K–Akt signaling pathway was predicted to be related to emergency response to diarrhea. Phosphoinositide 3-kinase is located upstream of the PI3K–Akt signaling pathway and is responsible for activating Akt; the activated Akt continues to phosphorylate downstream molecules (49). *Phosphatase and tensin homolog*, which is a core molecule downstream of the PI3K–Akt pathway (39), acts as a negative regulator of the pathway by inhibiting *PI3K* and *Akt* genes (50). The present study showed that the expression levels of *PI3K* and *Akt* were evidently downregulated; this may be due to the inhibition of *PI3K* and *Akt* genes in stress-induced diarrhea mice. Simultaneously, the phosphorylation of *PI3K* and *Akt* in the body was reduced as well. After the administration of QJC, the mRNA expression levels of *PI3K* and *Akt* in the small intestine of mice increased significantly (*p* < 0.05); we speculate that *PI3K* and *Akt* gradually returned to normal levels after the mice were given drugs, with phosphorylation of *PI3K* and *Akt* tending to be normal (30, 51). The *PTEN* expression was increased significantly under the induction of serotonin hydrochloride, but after QJC administration, the expression decreased; this is speculated to be caused by the normalization of phosphorylation of *PI3K* and *Akt* after QJC administration. Studies have shown that the expression of *Akt* was significantly decreased in mice with IBS (38). Interestingly, the active ingredients quercetin, luteolin, and kaempferol of QJC may also act as selective aryl hydrocarbon receptor (AHR) modulators in the AHR signaling and trigger an anti-stress diarrhea effect (52). The study shows that AHR can protect the intestine from inflammation by maintaining the homeostasis of intestinal stem cells and the integrity of the intestinal barrier (53). Aryl hydrocarbon receptor can also be used as a regulatory node in enteric neurons, which can maintain intestinal homeostasis and health by adjusting the environment of the intestinal cavity and the intestinal nerve circuit (54). Notably, the activation of AHR can also inhibit PTEN while inducing Akt (55), and our research also reports a similar result.

According to the report, quercetin is a ubiquitous bioactive flavonoid and a dietary component that can regulate physiological function through the effective expression of *PI3K, Akt*, and *PTEN* governed by the PI3K–Akt signaling pathway (56). Luteolin protects against HgCl_2_-induced cardiac damage by activating PI3K/Akt/Nrf2 signaling pathway (57). Using kaempferol to treat A549 cells can upregulate the PTEN protein and reduce p-PI3K and p-Akt protein, thus ultimately treating lung cancer by affecting the PI3K–Akt pathway (58). Scutellarein treatment can reduce the IL-1β-induced increase in IL-6 expression. Protein expression levels of Akt, phosphorescent p-Akt, mammalian target of rapamycin (mTOR), and p-mTOR in the PI3K/Akt/mTOR signaling pathway were decreased in the IL-1β-induced SW1353 cells following scutellarein treatment of osteoarthritis (59). Evidently, these four compounds are all flavonoids, a class of secondary metabolites of polyphenols that are produced mainly in fruits and vegetables and are widely believed to have health-promoting effects. In recent years, numerous studies have proven that flavonoids can be used to treat gastrointestinal diseases (60–62). This effect may be through enzymatic hydrolysis of flavonoids in the intestines of animals and further absorption and metabolism in intestinal epithelial cells or liver. In addition, flavonoids can also pass through the small intestine to the colon as unabsorbed, unmetabolized flavonoid glycosides or as flavonoids metabolized into conjugates in bile. Under the action of intestinal microbial enzymes, they are further metabolized into various ring fission products and finally exert biological functions. Moreover, the transformation into isoflavone may also be one of the ways of the antidiarrheal effect of flavonoids in the body (63).

In our study, we hypothesized that animals suffering from weaning stress-induced diarrhea can be effectively treated with QJC and its active ingredients, which work by affecting *PI3K, Akt*, and *PTEN* expression in the PI3K–Akt signaling pathway. The results of our study clearly proved our conjecture.

## Data Availability Statement

The original contributions presented in the study are included in the article/Supplementary Material, further inquiries can be directed to the corresponding author/s.

## Ethics Statement

The animal study was reviewed and approved by the Institutional Animal Care and Use Committee of Northeast Agricultural University (No. NEAUEC20).

## Author Contributions

YL designed the whole experiment. YZ directed the completion of the experiment. FY, EN, YW, XC, CL, and XW were supportive during the experiment. All authors contributed to the article and approved the submitted version.

## Funding

This work was supported by China Agriculture Research System of MOF and MARA, the Key Research and Development Program of Heilongjiang Province (Grant No. GA21B006).

## Conflict of Interest

JH and YL were employed by the company Harbin Lvda sheng Animal medicine Manufacture Co., Ltd. The remaining authors declare that the research was conducted in the absence of any commercial or financial relationships that could be construed as a potential conflict of interest.

## Publisher's Note

All claims expressed in this article are solely those of the authors and do not necessarily represent those of their affiliated organizations, or those of the publisher, the editors and the reviewers. Any product that may be evaluated in this article, or claim that may be made by its manufacturer, is not guaranteed or endorsed by the publisher.
