# Supplementary material for: Study on the Effective Material Basis and Mechanism of Traditional Chinese Medicine Prescription (QJC) Against Stress Diarrhea in Mice
Source: Front Vet Sci. 2021 Oct 4;8:724491. doi: 10.3389/fvets.2021.724491 (PMC8520981; doi:10.3389/fvets.2021.724491)

Supplementary Material

Study on the Effective Material Basis and Mechanism of Traditional Chinese Medicine Prescription (QJC) Against Stress Diarrhea in Mice

**Yuefeng Zhang^1,2^, Fei Yu^1,2^,Jingyou Hao^3^, ELIPHAZ NSABIMANA^1,2^,Yanru Wei^1,2^, Xiaohan Chang^1,2^, Chang Liu^1,2^, Xiaozhen Wang^1,2^, Yanhua Li^1,2,4*^**

^1^College of veterinary medicine, Northeast Agricultural University, Harbin,150030, China

^2^Heilongjiang Key Laboratory for Animal Disease Control and Pharmaceutical Development, Harbin, 150030, China

^3^Harbin Lvdasheng Animal medicine Manufacture Co., Ltd., Harbin,150030, China

^4^Harbin herb& herd Bio-Technology Co., Ltd., Harbin,150030, China

*** Correspondence:**Corresponding Author: Yanhua Li
liyanhua@neau.edu.cn

**Supplementary Table 1:** Bioactive compounds of QJC

| **NO.** | **Molecule ID** | **Molecule Name** | **DL** | **OB**  **(%)** |
| --- | --- | --- | --- | --- |
| HQ1 | MOL000211 | Mairin | 0.78 | 55.38 |
| HQ2 | MOL000239 | Jaranol | 0.29 | 50.83 |
| HQ3 | MOL000296 | Hederagenin | 0.75 | 36.91 |
| HQ4 | MOL000033 | (3S,8S,9S,10R,13R,14S,17R)-10,13-dimethyl-17-[(2R,5S)-5-propan-2-yloctan-2-yl]-2,3,4,7,8,9,11,12,14,15,16,17-dodecahydro-1H-cyclopenta[a]phenanthren-3-ol | 0.78 | 36.23 |
| HQ5 | MOL000354 | Isorhamnetin | 0.31 | 49.6 |
| HQ6 | MOL000371 | 3,9-Di-O-methylnissolin | 0.48 | 53.74 |
| HQ7 | MOL000374 | 5'-Hydroxyiso-muronulatol-2',5'-di-O-glucoside | 0.69 | 41.72 |
| HQ8 | MOL000378 | 7-O-methylisomucronulatol | 0.3 | 74.69 |
| HQ9 | MOL000379 | 9,10-Dimethoxypterocarpan-3-O-β-D-glucoside | 0.92 | 36.74 |
| HQ10 | MOL000380 | (6aR,11aR)-9,10-Dimethoxy-6a,11a-dihydro-6H-benzofurano[3,2-c] chromen-3-ol | 0.42 | 64.26 |
| HQ11 | MOL000387 | Bifendate | 0.67 | 31.1 |
| HQ12 | MOL000392 | Formononetin | 0.21 | 69.67 |
| HQ13 | MOL000398 | Isoflavanone | 0.3 | 109.99 |
| HQ14 | MOL000417 | Calycosin | 0.24 | 47.75 |
| HQ15 | MOL000422 | Kaempferol | 0.24 | 41.88 |
| HQ16 | MOL000433 | FA | 0.71 | 68.96 |
| HQ17 | MOL000438 | (3R)-3-(2-Hydroxy-3,4-dimethoxyphenyl) chroman-7-ol | 0.26 | 67.67 |
| HQ18 | MOL000439 | Isomucronulatol-7,2'-di-O-glucosiole | 0.62 | 49.28 |
| HQ19 | MOL000442 | 1,7-Dihydroxy-3,9-dimet-hoxy pterocarpene | 0.48 | 39.05 |
| HQ20 | MOL000098 | Quercetin | 0.28 | 46.43 |
| CQC1 | MOL001735 | Dinatin | 0.27 | 30.97 |
| CQC2 | MOL002714 | Baicalein | 0.21 | 33.52 |
| CQC3 | MOL002776 | Baicalin | 0.75 | 40.12 |
| CQC4 | MOL000359 | Sitosterol | 0.75 | 36.91 |
| CQC5 | MOL004004 | 6-OH-Luteolin | 0.28 | 46.93 |
| CQC6 | MOL000006 | Luteolin | 0.25 | 36.16 |
| CQC7 | MOL007796 | Stigmasteryl palmitate | 0.4 | 38.09 |
| CQC8 | MOL007799 | β-Sitosteryl palmitate | 0.4 | 30.91 |
| CQC9 | MOL002737 | Scutellarin | 0.24 | 18.97 |
| CQC10 | MOL007783 | Melampyroside | 0.8 | 57.5 |
| SJ1 | MOL000358 | Beta-sitosterol | 0.75 | 36.91 |
| SJ2 | MOL006129 | 6-Methylgingediacetate2 | 0.32 | 48.73 |
| SJ3 | MOL001771 | Poriferast-5-en-3beta-ol | 0.75 | 36.91 |
| SJ4 | MOL008698 | Dihydrocapsaicin | 0.19 | 47.07 |
| M | MOL000449 | Stigmasterol | 0.76 | 43.83 |

Note:DL, drug-likeness; OB, oral bioavailability.

**Supplementary Table 2**: The contents of active components in QJC were determined by HPLC

| **Ingredients** | **Regression Equations** | **R^2^** | **Linearity Range (mg/mL)** | **Content**  **(μg/g)** |
| --- | --- | --- | --- | --- |
| **Quercetin**  **Kaempferol**  **Luteolin**  **Scutellarin**  **Stigmasterol** | Y=34,364,384.7430x+1746.4279  Y=50,640,709.5949x+24,197.6866  Y=35,766,477.8962x+65577.7214  Y=38,652,626.6951x+1499.8358  Y=3,869,743.3063x+95,094.2388 | 0.9999  0.9992  0.9998  0.9995  0.9991 | 0.006-0.192  0.00125-0.04  0.00625-0.2  0.000391-0.0125  0.015625-0.5 | 4.91 ± 0.228  2.18 ± 0.237  25.58 ± 0.543  0.53 ± 0.026  145.72 ± 13.712 |

Note: Y,peak area; x, the concentration of the sample

**Supplementary Table 3**:The effect of QJC and its active ingredients on duodenum

| **Group** | **Villus height**  **(μm)** | **Crypt depth**  **(μm)** | **Villus height/**  **Crypt depth** | **Goblet cell count** |
| --- | --- | --- | --- | --- |
| **NC** | 381.52 ± 54.82^a^ | 74.51 ± 1.79^d^ | 5.13 ± 0.84^a^ | 32.67 ± 2.52^a^ |
| **MC** | 217.00±32.92^d^ | 126.09 ± 8.26^a^ | 1.73 ± 0.35^d^ | 16.00 ± 2.65^d^ |
| **PC** | 332.96 ± 62.03^bc^ | 84.88 ± 4.52^cd^ | 3.94 ± 0.79^b^ | 31.67 ± 2.08^a^ |
| **QJC** | 338.81 ± 27.75^bc^ | 94.79 ± 4.33^bc^ | 3.58 ± 0.32^bc^ | 28.00 ± 2.00^ab^ |
| **Quercetin** | 299.55 ± 60.51^bcd^ | 103.18 ± 11.90^b^ | 2.97 ± 0.83^bc^ | 25.00 ± 2.65^bc^ |
| **Kaempferol** | 289.93 ± 21.22^cd^ | 94.82 ± 8.92^bc^ | 3.08 ± 0.44^bc^ | 23.33 ± 3.05^bc^ |
| **Luteolin** | 257.20 ± 66.16^cd^ | 88.46 ± 2.42^c^ | 2.91 ± 0.73^bc^ | 21.00 ± 3.00^c^ |
| **Stigmasterol** | 316.34 ± 45.05^bc^ | 102.21 ± 4.32^b^ | 3.11 ± 0.57^bc^ | 23.33 ± 1.53^bc^ |
| **Scutellarein** | 286.26 ± 36.55^cd^ | 100.44 ± 5.63^b^ | 2.85 ± 0.32^c^ | 24.67 ± 1.15^bc^ |

Note:a-d indicate significant differences at P<0.05.

**Supplementary Table 4**:The effect of QJC and its active ingredients on jejunum

| **Group** | **Villus height**  **(μm)** | **Crypt depth**  **(μm)** | **Villus height/**  **Crypt depth** | **Goblet cell count** |
| --- | --- | --- | --- | --- |
| **NC** | 236.41 ± 7.32^a^ | 80.46 ± 4.71^c^ | 2.95 ± 0.25^a^ | 53.67 ± 1.53^a^ |
| **MC** | 93.38 ± 9.55^f^ | 105.49 ± 2.76^a^ | 0.89 ± 0.11^f^ | 32.33 ± 2.08^e^ |
| **PC** | 212.48 ± 16.28^ab^ | 90.05 ± 0.83^b^ | 2.36 ± 0.17^b^ | 46.67 ± 2.52^bc^ |
| **QJC** | 197.41 ± 13.22^bc^ | 94.76 ± 3.07^b^ | 2.08 ± 0.07^bc^ | 50.33 ± 4.04^ab^ |
| **Quercetin** | 166.48 ± 15.20^d^ | 94.15 ± 2.79^b^ | 1.77 ± 0.13^d^ | 43.00 ± 2.00^cd^ |
| **Kaempferol** | 180.53 ± 17.04^cd^ | 94.33 ± 2.95^b^ | 1.92 ± 0.24^cd^ | 49.66 ± 1.53^ab^ |
| **Luteolin** | 120.84 ± 7.49^e^ | 93.75 ± 4.23^b^ | 1.29 ± 0.04^e^ | 40.67 ± 0.58^d^ |
| **Stigmasterol** | 112.66 ± 5.77^ef^ | 92.30 ± 1.23^b^ | 1.22 ± 0.05^e^ | 40.67 ± 3.21^d^ |
| **Scutellarein** | 136.76 ± 28.06^e^ | 95.58 ± 3.49^b^ | 1.43 ± 0.30^e^ | 42.67 ± 3.06^cd^ |

Note:a-f indicate significant differences at P<0.05.

**Supplementary Table 5**:The effect of QJC and its active ingredients on ileum

| **Group** | **Villus height**  **(μm)** | **Crypt depth**  **(μm)** | **Villus height/**  **Crypt depth** | **Goblet cell count** |
| --- | --- | --- | --- | --- |
| **NC** | 195.69 ± 15.81^a^ | 43.85 ± 3.92^e^ | 4.49 ± 0.54^a^ | 59.33 ± 1.53^a^ |
| **MC** | 120.48 ± 4.43^c^ | 73.02 ± 4.87^a^ | 1.66 ± 0.15^c^ | 16.67 ± 3.21^f^ |
| **PC** | 139.51 ± 7.91^bc^ | 49.91 ± 3.86^de^ | 2.82 ± 0.36^b^ | 49.33 ± 2.08^bc^ |
| **QJC** | 144.57 ± 10.54^bc^ | 55.58 ± 1.26^cd^ | 2.60 ± 0.20^b^ | 50.33 ± 5.51^b^ |
| **Quercetin** | 153.36 ± 15.05^b^ | 64.09 ± 2.48^b^ | 2.39 ± 0.16^b^ | 47.33 ± 1.53^bcd^ |
| **Kaempferol** | 144.70 ± 28.72^bc^ | 62.75 ± 4.05^b^ | 2.32 ± 0.53^b^ | 44.00 ± 4.36^bcde^ |
| **Luteolin** | 155.57 ± 28.17^b^ | 65.04 ± 2.27^b^ | 2.39 ± 0.43^b^ | 40.67 ± 3.21^de^ |
| **Stigmasterol** | 154.02 ± 12.39^b^ | 59.88 ± 4.10^bc^ | 2.59 ± 0.37^b^ | 39.00 ± 4.36^e^ |
| **Scutellarein** | 149.62 ± 11.69^bc^ | 63.73 ± 2.97^b^ | 2.35 ± 0.19^b^ | 42.67 ± 4.93^cde^ |

Note:a-f indicate significant differences at P<0.05.

**Supplementary Fig. 1**:The standard and sample peak of active components. (A) The standard peak of quercetin. (B) The standard peak of kaempferol. (C) The standard peak of luteolin. (D) The standard peak of scutellarin. (E) The standard peak of stigmasterol. (F) The sample peak of QJC (quercetin and kaempferol). (G) The sample peak of QJC (luteolin). (H) The sample peak of QJC (scutellarin). (I) The sample peak of QJC (stigmasterol).


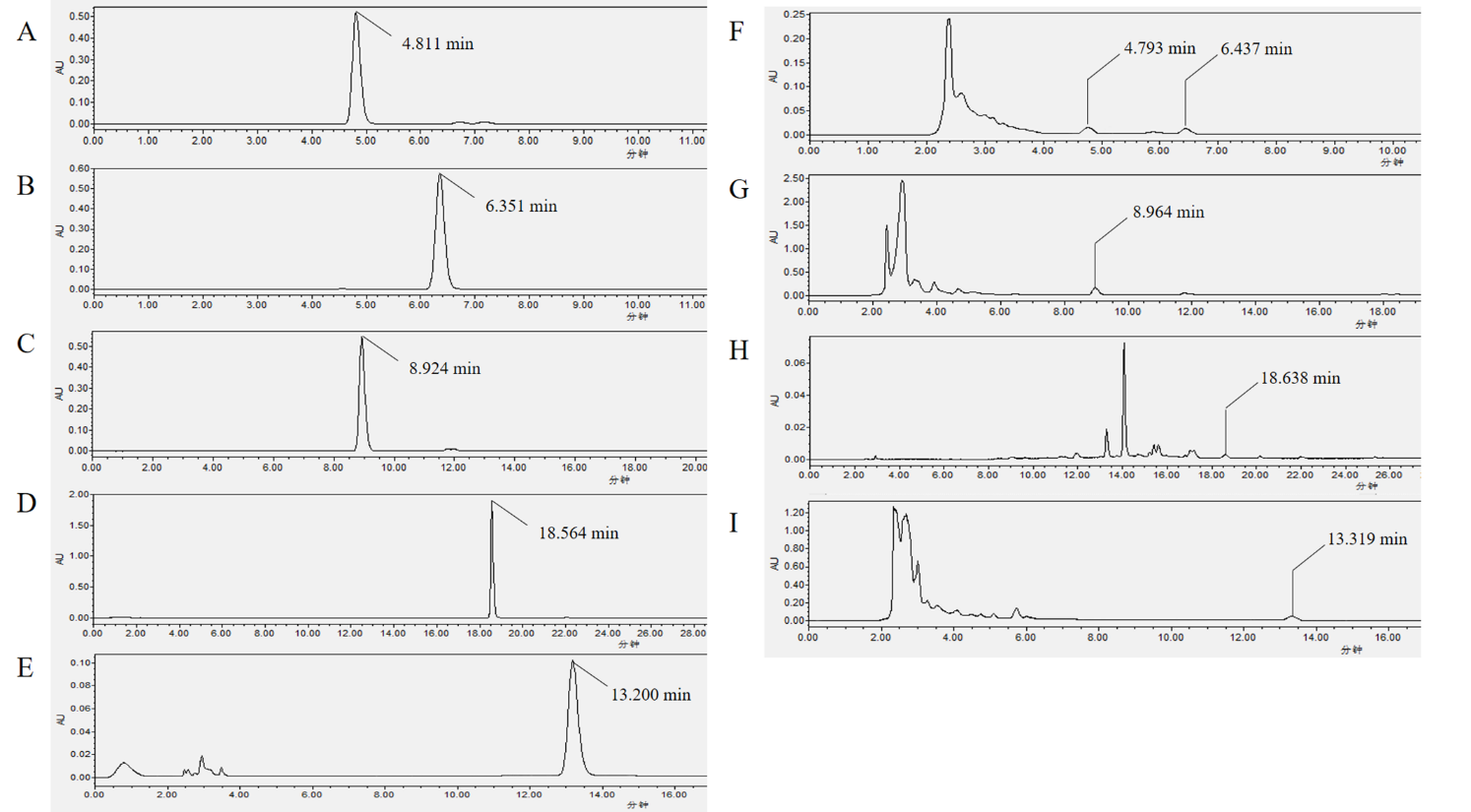

Supplement: Supplementary file 1 [file Data_Sheet_1.docx]
